# Language and Arithmetic: A Failure to Find Cross Cognitive Domain Semantic Priming Between Exception Phrases and Subtraction or Addition

**DOI:** 10.3389/fpsyg.2018.01524

**Published:** 2018-08-23

**Authors:** Golnoush Ronasi, Martin H. Fischer, Malte Zimmermann

**Affiliations:** ^1^Department of Linguistics, University Potsdam, Potsdam, Germany; ^2^Division of Cognitive Sciences, University Potsdam, Potsdam, Germany

**Keywords:** cross-domain priming, language, arithmetic, information integration, cognitive module

## Abstract

We examined cross-domain semantic priming effects between arithmetic and language. We paired subtractions with their linguistic equivalent, exception phrases (EPs) with positive quantifiers (e.g., “everybody except John”) while pairing additions with their own linguistic equivalent, EPs with negative quantifiers (e.g., “nobody except John”; Moltmann, 1995). We hypothesized that EPs with positive quantifiers prime subtractions and inhibit additions while EPs with negative quantifiers prime additions and inhibit subtractions. Furthermore, we expected similar priming and inhibition effects from arithmetic into semantics. Our design allowed for a bidirectional analysis by using one trial's target as the prime for the next trial. Two experiments failed to show significant priming effects in either direction. Implications and possible shortcomings are explored in the general discussion.

## Cross cognitive domain semantic priming: language and arithmetic

Whether different brain functions are strictly separated modules or have some overlap is of importance for our understanding of brain mechanisms since if module are strictly domain-specific and informationally encapsulated, they cannot transfer information between each other and therefore limit processing accuracy (Fodor, 1983; Bassok et al., 2008). Pinker (1997) also supports modularity, stating that the mind consists of different modules or “mental organs” each of which is specialized in a specific topic. He further explains that each module's specialty is defined by our genetics based on natural needs through human evolution. Chomsky (1980) suggests that language, from acquisition to development, is controlled by a specific module separated from other mental components. He believes that, similar to other organs of the human body, the brain is also structured and differentiated rather than being a functionally holistic organ.

Recent literature conflicts with the strictly modular view of the mind. Different cognitive domains in the brain may in fact be inter-connected (Patel, 2003, 2008; Van de Cavey and Hartsuiker, 2016). While some neuropsychological studies on aphasia and amusia patients show double dissociations between different cognitive domains such as language and music, neuroimaging findings show neural overlap when healthy people process linguistic and musical syntax (Patel, 2008). The above-mentioned conflicts of evidence led Patel to distinguish “knowledge representation” from “processing” systems. His hypothesis, known as Shared Syntactic Integration Resource Hypothesis (SSIRH), states that music and language each have their domain-specific syntactic “representation.” However, these representations depend on overlapping resources for “processing” the syntactic information (Patel, 2003). Thus, SSIRH explains cases of patients with selective amusia or aphasia by assuming that the deficiency is in the domain-specific representation and not in the shared processing resources.

Two widely used methods to tackle the above issue are *interference paradigms* and *priming paradigms*. Studies employing interference paradigms usually have participants simultaneously process stimuli from both domains. During critical trials, the stimuli from one domain include unexpected elements. Hypothetically if the two domains share the same network, the unexpected element from one domain will interfere with the participants' processing of the stimuli from the other domain as well. This will reflect in different patterns of reaction time (Van de Cavey and Hartsuiker, 2016). Priming, on the other hand, is a method where stimuli from one domain are followed by stimuli from the other domain. If the two domains share a network, then the former primes the latter, manifesting in a shorter reaction time (Van de Cavey and Hartsuiker, 2016).

Focusing on the priming approach, Scheepers et al. (2011) showed how the structure of a correct mathematical equation can manipulate people's choice of relative-clause attachment in completing a sentence. During the experiment, they presented participants one of the following mathematical structures: high attachment 80-(9 + 1) × 5 or low attachment 80-9 + 1 × 5. Later, participants received an open-ended sentence such as “*The tourist guide mentioned the bells of the church that….,”* and had to complete this sentence. Interestingly, the structure of the correctly solved equations primed the structure of the following sentence completion. Thus, the choice of whether the participants would attach the complement of the sentence to “*bells of the church”* (high attachment) or “*the church”* (low attachment) depended on the structure of the priming equation. The results demonstrate clear cross-domain structural priming from arithmetic to language. Scheepers and Sturt (2014) replicated this finding with a different task where participants solved left-branching or right-branching equations as well as rating left- or right-branching compounds of adjective-noun-noun on their sensicality. In order to test the effect of the priming direction, the experiment came in two versions; one with arithmetic stimuli as primes and the other one with linguistic stimuli as primes. Remarkably, structural priming was observed in both directions.

Most recently, Van de Cavey and Hartsuiker (2016) argued in favor of a domain-general structural processing mechanism by showing evidence of cross-domain and within-domain structural priming for language, music, and arithmetic. They primed participants' responses on different language tasks by arithmetic, musical, and other linguistic primes. Furthermore, by experimenting with non-hierarchically ordered color sequences, they showed that the displayed priming effect is necessarily a result of processing hierarchical dependencies and not just linear orders.

All of the above literature supports the existence of shared resources for syntactic processing, thus opposing the stronger version of brain modularity. According to this view, although each cognitive domain requires domain-specific syntactic representations, different domains still overlap in processing mechanisms involving integrating these representations. However, some questions still remain. Do these domains only integrate on the syntactic level? Or do they also integrate semantic information? For instance, can a sentence's semantic features prime semantic concepts in other cognitive domains, such as arithmetic? Whether SSIRH is limited to syntax or it is extendable to other language domains such as semantics can shed light on the extent of brain modularity.

Studies using semantic priming have shown similar results for integrating semantic and arithmetic information. Semantic priming is a technique analogous to the structural priming technique discussed in the previous section. The two methods differ with regard to the level at which the prime manipulates participants' responses. Bassok et al. (1998) showed that people's knowledge of semantic categorization affects their arithmetic performance. One of their first studies defines the concept of alignment between semantic relations and arithmetic operations. According to their definition, co-hyponyms or categorically related words (e.g., *roses* and *daisies*) form a semantic relation aligned with addition, while the functionally related words (e.g., *roses* and *vases*) form a semantic relation aligned with division. Further experiments (Bassok et al., 2008) suggest that arithmetic knowledge should not be separated from the other types of conceptual knowledge. The authors presented the participants with pairs of object words followed by two cue numbers presented with the artithmetic plus (+) sign in between. The participants' task was to decide whether a single digit presented after the cue numbers matched one of the cue numbers. For instance, they presented participants with 3 and 4 as the two cue numbers, and later asked them whether 7 was one of the cue numbers. Such experiments typically show a *sum effect*, i.e., participants show longer reaction time for rejecting sum targets than non-sum targets, indicating automatic addition.

Neuroimaging research by Guthormsen et al. (2015) confirms the conceptual integration of arithmetic operations with semantic categories. Their participants were presented with semantically aligned and misaligned arithmetic problems such as 6 roses + 2 tulips = ? vs. 6 roses + 2 vases = ?. Only during addition problems with misaligned objects, the second object label (here: vases) induced an N400 effect which is an electrophysiological response to semantic mismatch. Moreover, when participants were requested to judge the correctness of semantically aligned or misaligned problems a P600 effect was observed in reaction to mathematically correct but semantically misaligned problems. Since both N400 and P600 are signs of conceptual disruption, these experiments demonstrate how semantic categories are integrated online with arithmetic knowledge (Guthormsen et al., 2015).

Unlike addition and division problems, so far subtraction problems have not been used to show comparable cross-domain semantic priming between arithmetic and language. Reviewing the semantic literature, the closest equivalent of subtractions in language seem to be exception phrases (EPs) where the verb phrase is applied only to a subset of a group and not to the whole group (e.g., *every woman except Mary is dancing*). The construction of an EP has three main components: EP-associate, exception expression, and EP-complement. While the EP-associate is the noun phrase or quantifier which represents the bigger set that the exception phrase associates with, the EP-complement is what is excluded from the bigger set so that the sentence is true. For instance, in the sentence *every woman except Mary is dancing, every woman* is the EP-associate, *except* is the exception expression and *Mary* is the EP-complement.

Different semantic definitions have been proposed and employed for EPs throughout the years. The most well-known approaches are the ones proposed by Hoeksema (1987), von Fintel (1993), and Moltmann (1995). These approaches differ in several ways. Yet, all of them have in common that they consider EPs as subtractions in nature. Hoeksema (1987) defines EPs as language functions which subtract a set of elements from the universe of the sentence; see also Moltmann (1995). Later, von Fintel (1993) confirms that EPs are semantically equivalent to subtractions. He explains that a sentence containing an EP is true only if the EP-complement is the smallest set for which its subtraction from the EP-associate's domain leaves the sentence true (von Fintel, 1993). Moltmann (1995) also notes that EPs may act as additions or subtractions semantically, depending on the EP-associate. If the EP-associate is a negative universal quantifier (e.g., *nobody*), the EP-complement will be considered a subset that is added to the EP-associate, and therefore exception behaves as addition. For instance, the sentence *nobody except John goes to the class* means that John is the only one who does go to the class. Therefore, *John* denotes a set which is added to the zero intersection denoted by *nobody* and goes to class. However, according to Moltmann (1995) if the EP-associate is a positive universal quantifier such as *everybody*, the EP-complement has to be subtracted from the EP-associate. Considering the example *everybody except John goes to the class, one* understands that John is the only one not going to the class. In other words, John is subtracted from the group of people targeted by *everybody*, who are going to the class. Putting together the different approaches, one can conclude that all three analyses agree that EPs with positive quantifiers are the linguistic equivalent of arithmetic subtractions.

Moltmann (1995) identifies three conditions that EPs have to meet in order to be acceptable: the negative condition, the quantifier constraint, and the condition of inclusion. An EP meets the negative condition when applying the verb phrase to the EP-complement reverses the truth value of the sentence from true to false. For instance, if *everybody except John goes to the class* is true, the application of the meaning of the VP *goes to class* to the meaning of the EP-complement *John* will result in a false statement (*John goes to the class*). The quantifier constraint states that the EP-associate should denote a positive or negative universal quantifier, such as *all students* or *no student*, respectively. And lastly, according to the condition of inclusion, the EP-complement should belong to the set denoted by the EP-associate. Of these conditions, the *condition of inclusion* is of greatest interest to this study. As mentioned above, the condition states that the EP-complement has to be included in the larger set denoted by the EP-associate. It was also explained earlier that Bassok introduced the concept of alignment, where the sum effect only happens when the priming words are semantically aligned with addition. This is the case with words that are co-hyponyms, i.e., when they are members of the same larger set. Considering Bassok's results, which support the existence of a link between semantic knowledge and the arithmetic memory network, it is plausible to explain the driving force of the above condition in terms of alignment of semantic categories and additions, keeping in mind that subtractions are the inverse act of additions. This implies that additions and subtractions as their inverse function should only be licit when the subtracted or added set is categorically related to the other set. Therefore, in exception sentences with negative universal quantifiers, it is only possible to exclude or add a subset of a group to the rest of the group. Similarly, in exception sentences with positive universal quantifiers, it is only possible to exclude or subtract a subset of a group from the rest of the group. Overall, it seems reasonable to take exception phrases with positive universal quantifiers as the linguistic equivalent of subtractions, whereas exception phrases with negative universal quantifiers form the equivalent of additions.

Here, we are interested to further investigate the extent to which different cognitive domains share a semantic processing mechanism. For this, we are looking for bi-directional cross cognitive domain semantic priming between arithmetic and language. More precisely, we are inspecting whether sentences with an Exception Phrase and subtraction or addition arithmetic problems prime or inhibit each other.

The present study includes two experiments. In the first experiment we expect to see priming effects between exception sentences and arithmetic subtractions. Additionally, we expect exception sentences to inhibit addition problems. Based on evidence from numerical cognition research, we expect additions to be generally faster than subtractions (e.g., Kamii et al., 2001), therefore we do not necessarily expect faster reaction times for subtractions compared to additions following exception sentences. Given that this is the first experiment investigating the potential semantic relationship between EPs and arithmetic, the current study is a big step in comprehending whether there is a cross-domain priming effect from one stimulus type to another in either direction.

## Experiment I

### Method

The senior author (MHF) ensured that the study was carried out in accordance with the guidelines of the British Psychological Society (2000), including written informed consent and confidentiality of data as well as personal conduct.

#### Participants

Thirty two native speakers of German (24 female) aged between 19 and 64 years old (mean = 37.22, sd = 13.64) participated in the experiment. Participants' educational backgrounds varied from languages and literature to physics and IT. Students were compensated with ½ participation credit. Signed consent was obtained from all participants.

#### Materials

##### Device and software

The experiment was coded in Python and run in *Expyriment*, an open source, python-based software developed for designing experiments (Krause and Lindemann, 2014). It was presented to participants on a 15.6 inch Toshiba satellite L50-B-2CC laptop screen operating on windows 10 in a quiet room. Stimuli were displayed in white free mono font on a black screen. The font size differed depending whether it was a sentence (size: 40 points), an equation (size: 60 points) or a response feedback (size: 80 points and in red). Participants' responses were recorded via key press on a computer keyboard. We counterbalanced the keys due to the Operational Momentum (OM) effect. According to evidence from Spatial-Numerical Association of Response Code (SNARC) and OM effects, participants' reaction time to subtractions and additions is tied to direction in the way that subtractions are biased leftward and additions are biased rightward (Pinhas and Fischer, 2008). Hence, we swapped the key mapping between participants so that half of the participants were trained to press “L” for meaningful sentences and correct equations and “S” for meaningless sentences and incorrect equations while the other half were trained for the reverse mapping.

#### Stimuli

##### Sentences

Based on 30 meaningful exception sentences, a total of 120 German sentences were created to fit into 4 different groups; meaningful exception, meaningless exception, meaningful non-exception, and meaningless non-exception (see Supplementary Material). While 60 sentences consisted of an EP, the rest were non-exception, general statements. In both the exception and the non-exception group, half of the sentences were meaningful while the other half featured a semantic anomaly, making them non-sensical and meaningless upon encountering the last word (e.g., Jede Kuh außer Linda frisst Sonne, “Every cow except Linda eats sun”). A sentence is decided to be meaningful if it is not immediately falsified against the actual world based on the linguistic meaning of the sentence alone. To achieve a comparable reading time and parsing difficulty, it was important to have sentences with similar syntactic and semantic features as well as a similar length. Therefore, syntactic and semantic features were selectively controlled throughout the sentences. The controlled syntactic features included tense, movements, sentence structure, number of syntactic nodes and word number, while semantically we controlled and restricted the choice of the exception expression and the EP-associate.

All sentences were declarative, created in simple present tense without syntactic movements. We created all sentences based on a specific syntactic tree with 5 syntactic nodes. This is due to research by Chang and Kuo (2009) showing that the height of a syntactic tree is a reflection of sentence complexity. This is also supported by a study which reveals a correlation between EFL (English as a Foreign Language) learners' reading time and the number of syntactic nodes in a sentence (Chang and Kuo, 2009). Additionally, all sentences had exactly 6 words. Exceptions regarding syntactic nodes and word number occurred when the last word of the sentence needed an overt article, e.g., *Jeder Schriftsteller ist chaotisch im Arbeitszimmer* (Every writer is messy in the office) vs. *Alle Freundinnen besitzen Autos für die Arbeit* (All friends own cars for work). Finally, words specific to a regional dialect which could have caused delays in participants' reading time were avoided. Beside the syntactic factors mentioned above, two semantic factors were also taken into consideration while creating the sentences: the exception word and the EP-associate. According to Moltmann (1995), sometimes an EP operates at the level of implied semantic structuring. The EP- complement (*the door*) and the EP-associate (*the house*) in the following sentence: “*except for the door, John painted the house”* act as an example for this type of EP. In these cases the EP-complement (*the door*) is semantically relevant to the EP-associate (*the house*) but this relevance is not as explicit as in cases where the EP-complement and the EP-associate are from the same semantic categories (e.g., *the chair* and *the furniture* in *except for the chair, John painted the furniture*).

Interestingly, in some languages, such as German, this also affects the choice of EP as some exception words do not work at the level of implications while some others do. In German, the exception phrase *bis auf* works better than *außer* in such sentences, e.g., *Der Raum war leer bis auf einen Stuhl* “Every room was empty except for a chair”(Moltmann, 1995). However, in the sentence *Jeder Mann bis auf/außer Hans* “Every man except Hans”, where the EP-complement (*Hans*) is a subset of the EP-associate (*Men*), the two exception phrases may be used interchangeably. This can potentially demonstrate an underlying difference between these two types of exception sentences. Therefore, to assure that all our sentences are semantically similar to each other, we decided to only use exception sentences with “außer.”

The last component to control for was the EP-associate. As mentioned earlier in the literature, the nature of EPs with negative quantifiers is equivalent to that of additions, while the nature of those with positive quantifiers is equal to that of subtractions (Moltmann, 1995). Hence, to keep the exception sentences homogeneous, all our sentences started with one of the positive universal quantifiers, *jeder* or *alle* “every/all.”

To create the sentence stimuli, 30 meaningful exception sentences were created following the designed sentence structure. The next step was to create meaningful non-exception sentences based on our meaningful exception sentences. In order to do so, the exception word and the EP-complement were removed from each sentence; moreover, to keep the word number equal, a two-word adverbial phrase was added to the end of the sentence. In a few cases where an adverbial phrase was not possible, an NP-modifier was added (e.g., *Jede Schülerin trägt Hosen mit Knöpfen*). Finally, the last word of each sentence was changed appropriately in order to create meaningless analogs. Table 1 shows the overall sentence structures across the conditions. All sentences are presented in Supplementary Material.

**Table 1:** Stimuli sentence structure.

**Structure**	**EP associate**		**EP complement**		**VP**	
**Word items**	**Jede/Alle**	**Noun**	**außer**	**Noun**	**Verb**	**Comp**.
Meaningful Exception	Jede	Kuh	außer	Linda	frisst	Gras
	‘Every	cow	except	Linda	eats	grass'
Meaningless Exception	Jede	Kuh	außer	Linda	frisst	Sonne
	‘Every	cow	except	Linda	eats	sun'
			**VP**		**AdvP**	
Meaningful Non-exception	Jede	Kuh	frisst	Gras	auf	der Weide
	‘Every	cow	eats	grass	on	the meadow'
Meaningless Non-exception	Jede	Kuh	frisst	Gras	Auf	dem Asphalt
	‘Every	cow	eats	grass	on	the asphalt

#### Equations

Besides sentences, 120 arithmetic equations (60 additions, 60 subtractions, half of each correct) were also created (see Supplementary Material). To avoid parity matching, all incorrect results were generated by adding ± 2 to the correct results. Problem size was also controlled (following Fayol and Thevenot, 2012). All equations were two-digit numbers ± a one-digit number with the carryover effect. In case of additions the operands were chosen in a way that the sums were all equal or larger than 25. To control for repetition priming, care was taken to avoid any inverse equations throughout the materials (i.e., presence of 28–9 results in the omission of its inverse addition “19 + 9”).

#### Design

The experiment followed a 2 × 2 × 2 (prime: exception vs. non-exception × meaning: meaningful vs. meaningless × task: subtraction vs. addition) design. All participants went through the same set of sentences and equations. However, to eliminate the random effects of sequential order of trials and conditions, and to ensure that the results are not bound to any specific prime-target pairing, different lists with same stimuli but different randomizations were created. During the experiment, each participant received a unique list. For the sake of consistency through the experiment and to ensure proper counterbalancing, in each list, the following pairing pattern was utilized. First of all, within each group of sentences (exception, non-exception, meaningful, meaningless) half of the sentences were paired with additions while the other half were paired with subtractions. Among the 15 equations paired with sentences from a specific sentence condition, 7 or 8 equations were correct and the other 8 or 7 equations were incorrect. This was counterbalanced across conditions and pairings (Table 2).

**Table 2:** Sentence - equation pairing.

**Sentence condition**	**Equation**	**Correctness**	**Sentence condition**	**Equation**	**Correctness**
30 Meaningful Exception	15 Addition	7 correct	30 Meaningful non-exception	15 Addition	8 correct
		8 incorrect			7 incorrect
	15 subtraction	8 correct		15 subtraction	7 correct
		7 incorrect			8 incorrect
30 Meaningless Exception	15 Addition	8 correct	30 Meaningless non-exception	15 Addition	7 correct
		7 incorrect			8 incorrect
	15 subtraction	7 correct		15 subtraction	8 correct
		8 incorrect			7 incorrect

First, the 32 lists were divided into 8 different groups where each group had a different randomization of conditions. This ensured that different conditions would follow each other and the results show a true effect of the same trial's prime and not the prime or target of a previous trial. Additionally, the sentences and equations were randomized within each of the mentioned condition groups to eliminate the effect of stimuli's sequential order. Ultimately, to avoid a potential pairing effect, the sentence-equation pairs were unique for each participant. At the end, each of the 32 different lists had different sequential ordering for conditions and trials, and unique sentence-equation pairs. Across all lists, each sentence was paired both with additions and subtractions.

#### Procedure

Participants were informed that they would see meaningful and meaningless sentences together with correct and incorrect equations. They were instructed to judge the stimuli on their meaningfulness or correctness as quickly and as accurately as possible by pressing the dedicated keys. The experiment started with a short practice round consisting of 12 trials. This was followed by the main experiment which lasted about 15–20 min depending on participants' speed and accuracy as they would receive a feedback after incorrect responses. During the main experiment each participant was presented with 120 trials going through 120 unique sentences and 120 unique equations. The practice round was a short version of the main experiment with different stimuli. Participants who still found the experimental task unclear could ask for clarification before proceeding with the main experiment. Questions were answered as long as it would neither reveal the purpose of the study nor create inconsistency within participants.

Each trial started with a sentence as the prime and ended with an equation as the target; both were presented centered on the screen. During each trial, participants were presented with a sentence, exception or non-exception, and they were requested to judge them as meaningful or meaningless by a key press. Once they pressed one of the two possible keys, the sentence would disappear and the target equation would immediately appear on the screen. Then, participants had to decide whether the equation result presented on the screen was correct or incorrect. They used the same keys for marking *correct* and *incorrect* that they had used for *meaningful* and *meaningless*, respectively. As for the response keys, the keys “L” and “S” were randomly selected as two keys sufficiently far apart from each other on the keyboard. The experiment allowed participants to press keys as soon as they had an answer after seeing the stimulus (prime/ target) on the screen. The program would measure the reaction time from the moment the stimulus was on the screen until the participant reacted to each stimulus by pressing a key. Therefore, the measure included participants' reaction times to the stimulus together with their reading times. Also, there was no time limit during which the participant had to respond. This means the stimuli stayed on the screen until one of the keys was pressed. This was to prevent rushing participants as each may have a different reading pace. It also helped removing trials which may show effects of tiredness or boredom. During the experiment, participants received a written verbal feedback right after making a mistake. The feedback appeared on the screen for 3 s. This ensured that the participants paid attention to the experiment and also helped them refine their understanding of the task if they were still unclear. Feedback itself was not an exclusion criterion for trials.

#### Data trimming

Prior to data analysis, a number of sentence-equation pairs and trials were removed from the data. It is important to mention, that as a first step of data trimming prior to data analysis in each direction, the raw data was converted to a horizontal table (i.e., each prime and target pair created a one-line trial in the data table). Therefore, each trial could be easily removed without changing the consequence of primes and targets within a single trial.

First, we removed one equation which was answered incorrectly by more than fifty percent of the participants. Any trial containing this item was removed due to the item's low accuracy rating across participants (item accuracy across participants = 47%, number of overall removed trials for this reason = 32). This caused removal of 0.83% of the original data. Second, to avoid inclusion of anticipatory guessing, any trials with prime or target reaction time shorter than 500 ms were removed from the remaining data. Given the limited duration of the cognitive priming mechanism of interest, we reduced variability by removing trials with prime or target reaction times longer than 6,000 ms (trimmed data: 252 trials, 6.57%). Lastly, for reaction time analysis we only included trials which elicited a correct target response (trimmed data: 305 trials, 7.09% of the original 8.58%). Overall, 15.34% of the original data were removed during data trimming.

### Results

All 32 participants passed an 80% accuracy threshold in judging both whether a sentence was meaningful (*mean* = *90.78%, sd* = *0.07)* and whether an equation was correct (*mean* = *91.42%, sd* = *0.05*). They were overall most accurate in judging meaningless non-exception sentences (*mean* = *95.5%, sd* = *0.21*) and least accurate in judging meaningful exception sentences *(mean* = *85.4%, sd* = *0.35*). According to our hypothesis and design, exception sentences and subtraction equations were congruent conditions, while exception sentences and addition equations were incongruent conditions. Expecting a priming effect for the congruent conditions (exception sentences with subtractions) and an inhibitory effect for the incongruent condition (exception sentences with additions), a linear model with multiple regressions was fitted on the target equation reaction times as a function of prime (see Table 3). The linear model was set to study the potential effects of meaning (meaningful vs. meaningless), prime (exception vs. non-exception), task (addition vs. subtractions) and their interactions. Aside from the task, no other factors including meaning or prime affected the target reaction times significantly, all *p*-values > 0.25. According to this test, there were no significant differences in participants' reaction times to equations depending on whether these were preceded by an exception or a non-exception sentence.

**Table 3:** Linear model results on equation reaction time.

**Effect**	**Estimate**	**SE**	***t***	***p***
Intercept	2,659.05	19.50	136.36	< 0.001^***^
Meaning	18.37	19.50	0.94	0.35
Prime	−15.39	19.50	−0.79	0.43
Task	115.89	19.50	5.94	< 0.001^***^
Meaning-Prime	2.07	19.50	0.10	0.91
Meaning-Task	−8.36	19.50	−0.43	0.67
Prime-Task	−8.14	19.50	−0.42	0.68
Meaning-Prime-Task	−22.53	19.50	−1.15	0.25

*** *Indicates significance level*.

**Figure 1:**
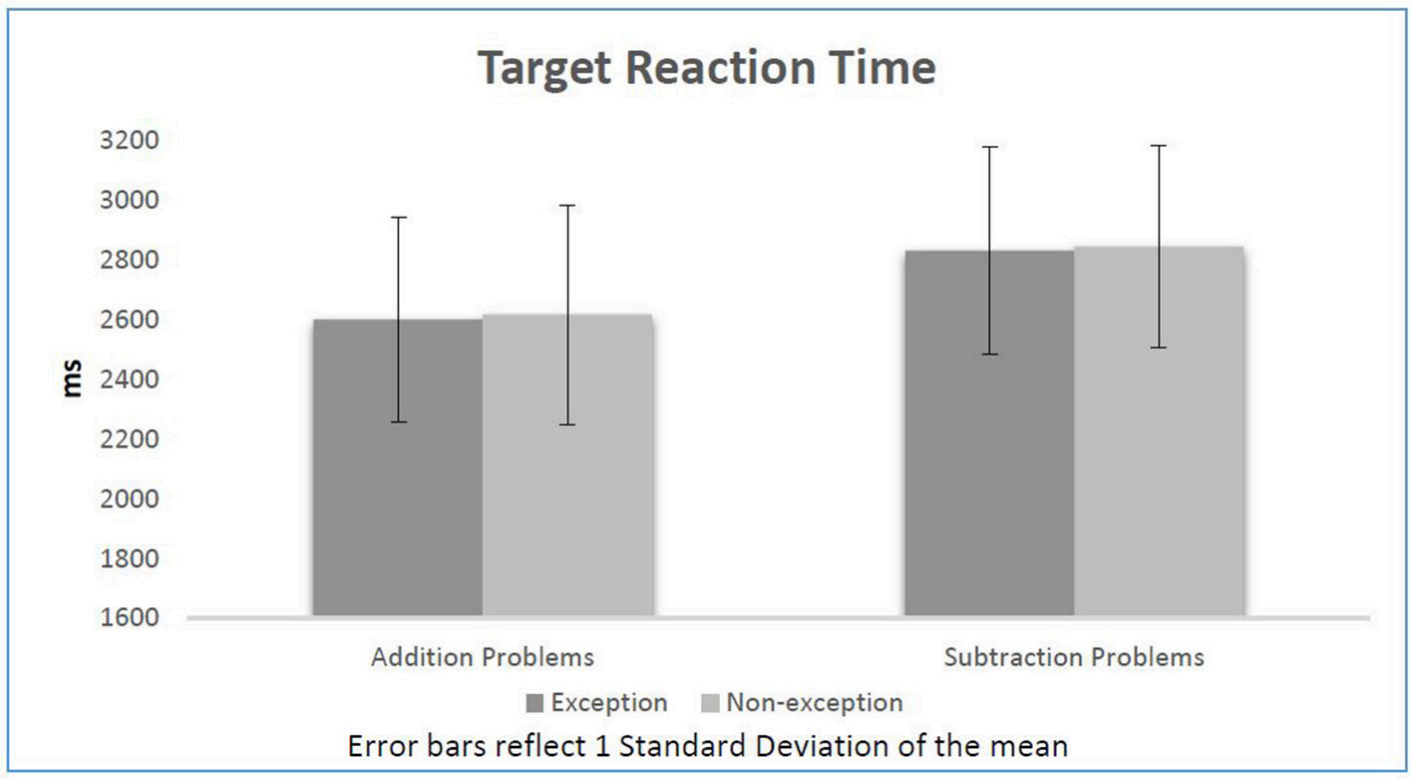
Target reaction time per condition.

To further confirm our results, we added paired *t*-tests after each linear model. According to the paired *t*-tests participants reacted significantly faster in response to additions (2,606 ms) in comparison with subtractions (2,836 ms), *t*_(31)_ = −5.46, *p* < 0.01. Even though most participants typically reacted faster to subtractions preceded by exception sentences (2,830 ms) than the ones preceded by non-exception sentences (2,843 ms), these differences were not statistically significant, *t*_(31)_ = −0.28, *p* = 0.78. Similarly, the data did not show any inhibition effect on additions. The difference of reaction time between additions following exceptions (2,600 ms) and additions following non-exceptions (2,615 ms) was not statistically significant, *t*_(31)_ = −0.33, *p* = 0.74. Information on reaction time per condition is shown in Figure 1.

Overall, the current experiment did not show significant priming of arithmetic by language. Only 6 participants showed both the priming and the inhibitory effects, while 15 participants only showed the priming effect for subtractions and 13 participants only showed the inhibitory effect for additions.

We also expected the subtraction problems to prime exception phrases of their following trials and therefore facilitate participants' judgment on them. In other words, we were expecting faster reaction times for exception sentences following subtractions than for the exception sentences following additions. In order to address this hypothesis, we analyzed the reaction times to sentences as a function of their preceding equation. Understandably, this only included sentences from the second trial to the last trial for each participant. The fitted linear model on the data revealed no significant effect of priming from the preceding equation, *p* = 0.94 (Table 4).

**Table 4:** Linear model results on arithmetic priming language.

**Effect**	**Estimate**	**SE**	**|*t*|**	***p***
Intercept	2,478.2	21.50	115.27	< 0.001^***^
Equation	2.20	30.55	0.07	0.94

*** *Indicates significance level*.

Additionally, we did a *t*-test on participants' reaction times on exception phrases after subtractions and additions. The *t*-test showed that the difference of reaction time between exception sentences preceded by subtractions (2,665 ms) and exception sentences preceded by additions (2,620 ms) is statistically non-significant, *t*_(31)_ = 0.37, *p* = 0.71. Finally, we determined whether our results are contaminated by a motor priming effect. Motor priming occurs when a key press is facilitated by being repeated. The effect was tested within trial and in both directions (i.e., sentence > equation for the first analysis and equation > sentence for the second analysis). We re-defined congruent and incongruent conditions based on whether the key response to the previous item was identical to the one to the current one but found no reliable difference, *p* > 0.15.

### Discussion

As expected by the literature, addition problems were judged faster than subtraction problems. However, the data failed to show a statistically significant priming effect between exception phrases and subtractions in either direction. Certain limitations could explain why this experiment failed to reveal the semantic priming effect between language and arithmetic. First of all, a crucial step in priming studies is to make sure that participants have processed the primes. Otherwise, one cannot expect a priming effect. This is the main reason why primes are usually followed by a comprehension task. The current study was based on the hypothesis that EPs will prime subtractions. However, the comprehension task following the primes had nothing to do with processing the EPs. Since the sentence anomaly was a semantic mismatch between the EP-associate and the sentence predicate, the exception word and the EP-complement did not play a role in determining the meaningfulness of the prime sentences. In practice, one could skip reading the exception word and the EP-complement, not processing the EP as a result, and yet answer the comprehension task correctly. Developing task-specific strategies usually happens when the same type of comprehension task repeats after each stimulus (Keating and Jegerski, 2015). Therefore, the non-significant results may be due to many participants skipping the EPs and reading only the phrases which are crucial to the comprehension task.

Furthermore, since the comprehension tasks were not addressing the primes directly, we decided to keep the trials with incorrect prime responses in the analysis. However, while those sentences may have initially primed the participants, the 3 s written verbal feedback after the incorrect response may have removed the priming effect in those trials.

Another issue could be task complexity. After the experiment, participants commented about the task's complexity while judging the sentences. Participants were requested to judge the meaningfulness of the sentences and although care was taken while creating the sentences so that semantic ambiguities do not affect participants' judgment, the task was still not as clear for participants as it should have been. Based on the comments, participants were not confident about their semantic judgements on some of the sentences. Some would argue that a sentence like *Everybody drinks milk* is meaningless since some people are allergic to milk, while some other would argue that a sentence like *All cows eat bags* may be considered meaningful but simply false. It is noteworthy that while most participants found it difficult to judge sentences, in most cases these sentences were not the same for all participants leading us to conclude that it was a more general issue than an issue with specific sentences. It seems like the materials were not suitable for helping participants dissociate meaningfulness from truth value.

A joint solution to both problems mentioned above, i.e., primes not being task crucial and the task being complex, is to have the semantic anomaly within the EP (e.g., *Every boy except the table*). This modification would make processing the EPs a requirement for responding to the comprehension task, while disambiguating and lowering the task's complexity. Additionally, we could have the EP-complement in the object position (e.g., *Every boy goes to school except the table, Jeder Junge geht zur Schule, außer dem Tisch*) so that the sentence ends with the anomaly. This would be to ensure that the reaction time is a better indicator of participants' reaction to the anomaly. Another factor which could potentially extract a statistically more significant effect is using stimuli which prime additions as well. The current design had exception sentences and non-exception sentences, the former acting as primes for subtractions and the latter acting as control. As mentioned earlier, exception sentences with negative universal quantifiers, such as *Nobody except John goes to the class*, act semantically as additions (Moltmann, 1995).

Our second experiment therefore implemented a design with EPs with positive universal quantifiers and EPs with negative universal quantifiers (e.g., *Every student except John* vs. *No student except John*) where the first group primes subtractions while second group primes additions. Priming additions as well as subtractions could potentially create a larger gap between the primed and blocked reaction times and therefore provide a statistically more significant result.

## Experiment II

Experiment II was designed to overcome the limitations of the linguistic stimuli of Experiment I. Therefore, the arithmetic stimuli, experimental device, design, and procedure remained the same as before. However, the linguistic stimuli were updated appropriately to (a) have the primes more crucial to the task, i.e., semantic anomaly happens within the EP (b) prime additions as well as subtractions to potentially achieve statistically more reliable results and (c) reduce the task ambiguity.

### Method

#### Participants

We recruited a more homogeneous group of participants for the second experiment. 32 native speakers of German (17 female) from the age of 18–33 years old (mean = 23.62, *sd* = 4.19) participated in the experiment. Participants' educational backgrounds varied from languages and literature to Chemistry and IT. Students received 1 participation credit. Signed consent was obtained from all participants.

### Materials

#### Sentences

A total of 120 German sentences were created to fit into 4 different groups; meaningful *Exception with Positive Universal Quantifier* (EPUQ), meaningless EPUQ, meaningful *Exception with Negative Universal Quantifier* (ENUQ) and meaningless ENUQ (see Supplementary Material). While 60 exception sentences consisted of a positive universal quantifier, the rest were created with a negative universal quantifier. In both groups, half of the sentences were meaningful while the other half featured a semantic anomaly, making them nonsensical and meaningless upon encountering the last word (e.g., Jeder Junge geht zur Schule, außer dem Tisch, “Every boy goes to school except the table”). It was important for the semantic anomaly to happen within the EP. Table 5 shows the overall sentence structures across the conditions. All sentences are presented in Supplementary Material.

**Table 5:** Stimuli Sentence Structure.

**Structure**	**EP associate**		**VP**		**EP complement**	
**Word Items**	**Jede/Alle/Kein**	**Noun**	**Verb**	**Comp**	**außer**	**Noun**
Meaningful EPUQ	Jedes	Kind	ist	hungrig	außer	Peter
	‘Every	kid	is	hungry	except	Peter'
Meaningful ENUQ	Kein	Kind	ist	hungrig	außer	Peter
	‘No	kid	is	hungry	except	Peter'
Meaningless EPUQ	Jedes	Kind	ist	hungrig	außer	dem Tisch
	‘Every	kid	is	hungry	except	the table'
Meaningless ENUQ	Kein	Kind	ist	hungrig	außer	dem Tisch
	‘No	kid	is	hungry	except	the table'

Overall, the experiment included 30 meaningful EPUQs, 30 meaningless EPUQs, 30 meaningful ENUQs and 30 meaningless ENUQs. Similar syntactic and semantic factors as in experiment I were controlled to achieve a comparable reading time and parsing difficulty. The sentences were initially created as meaningful EPUQ. Each sentence was later turned into a meaningful ENUQ by changing the universal quantifier from Alle/ Jede into Kein. As the final step, the last word of each sentence (e.g., the EP-complement) was changed to a word outside the semantic category of the EP-associate in order to create the meaningless counterparts.

##### Equations

The same 120 equations used in experiment I were utilized in Experiment II as well.

#### Design

Experiment II followed a 2 × 2 × 2 (prime: exception with PUQ vs. exception with NUQ × meaning: meaningful vs. meaningless × task: subtraction vs. addition) design similar to the experiment I. It was only the nature of the prime which had changed from exception vs. non-exception to exception sentences with PUQ vs. exception sentences with NUQ. The mentioned alteration would let us pair the EPUQs with subtractions and ENUQ with additions. The stimuli and the design experienced the same randomization procedure as in Experiment I.

#### Procedure

Experiment II followed the same procedure as Experiment I.

### Analysis

#### Data trimming

Based on inspection of the reaction time distribution for these modified linguistic materials, 440 of 3840 trials had prime or target reaction times more than 5,500 ms or < 500 ms and were removed (trimmed data: 11.46%). As in this experiment the task was directly on processing the prime, it made sense to only analyze the data when the participants had correctly processed and judged the prime. Therefore, we removed 78 trials where the participant had misjudged the prime (trimmed data: 2.3%). Furthermore, in reaction time analysis we also removed 215 trials where the target was incorrectly judged (trimmed data: 6.47%). Overall, for accuracy analysis and reaction time analysis we respectively excluded 518 and 733 trials or 13.49 and 19.09% of the original data during trimming.

### Results

Experiment II elicited higher prime accuracy within subjects (97.1%) compared to Experiment I (90.78%) showing the effect of stimuli improvement on reducing task ambiguity [*t*_(31)_ = −4.91, *p* < 0.0001]. In this experiment, we were expecting for the exception sentences with PUQs to prime subtractions and for the exception sentences with NUQs to prime additions. The effects were also expected to induce inhibition; i.e., EPUQs inhibiting additions and ENUQs inhibiting subtractions. A linear model fitted on the data to investigate the hypotheses above revealed no effect of prime on target reaction time. In fact once again, task is the only factor affecting participants' reaction times on target equations, *p* < 0.0001 (see Table 6).

**Table 6:** Linear model results on equation reaction time.

**Effect**	**Estimate**	**SE**	***t***	***p***
Intercept	2,648.63	17.76	149.17	< 0.001^***^
Meaning	−14.73	17.78	−0.83	0.41
Prime	17.98	17.78	1.01	0.31
Task	129.07	17.78	7.26	< 0.001^***^
Meaning-Prime	13.46	17.78	0.76	0.45
Meaning-Task	5.04	17.78	0.28	0.77
Prime-Task	−9.9	17.78	−0.55	0.58
Meaning-Prime-Task	8.3	17.78	0.47	0.64

*** *Indicates significance level*.

**Figure 2:**
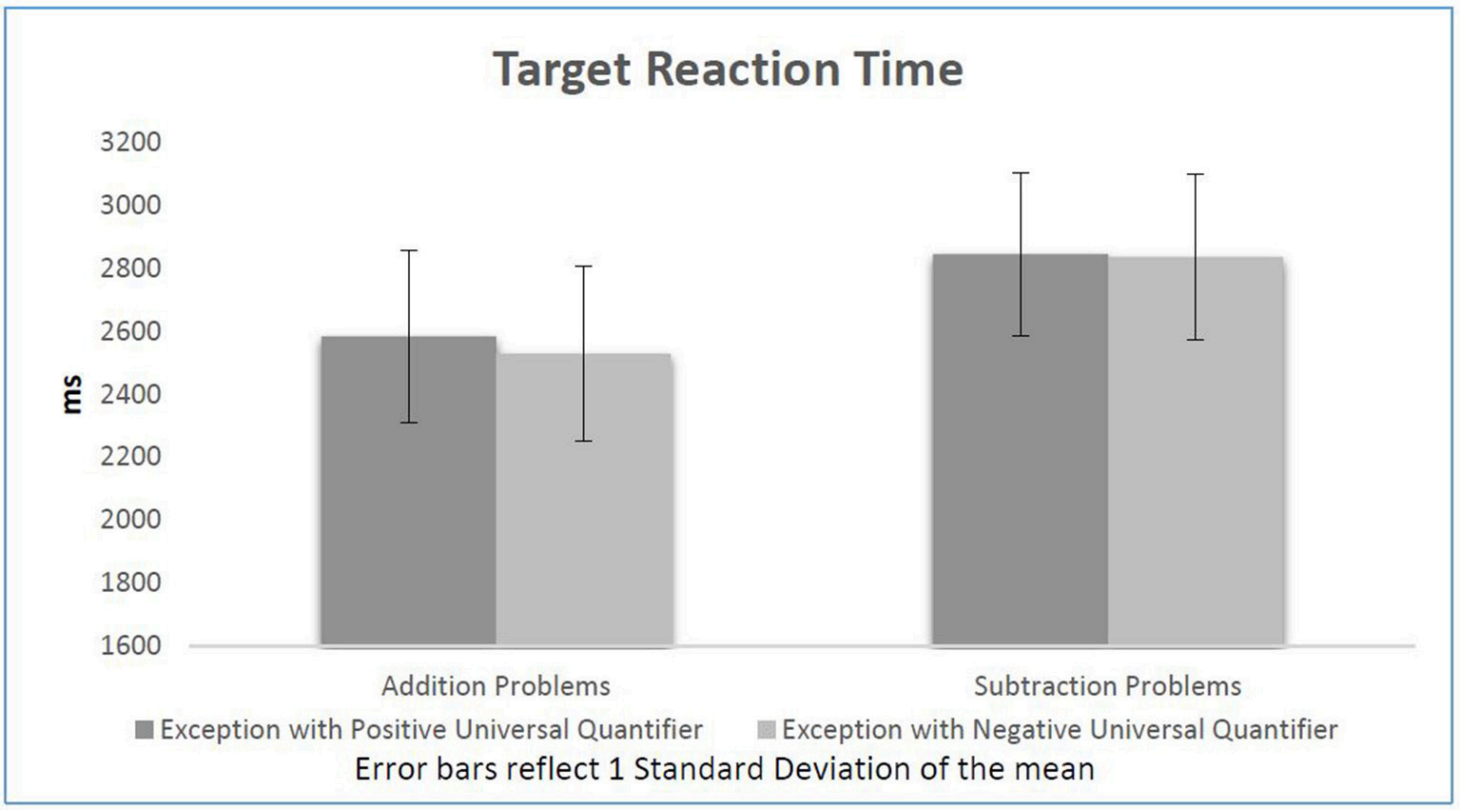
Target reaction time per condition.

Further paired *t*-tests also show that as expected by the literature and the previous experiment, participants generally reacted significantly faster in response to additions (2,557 ms) in comparison to subtractions (2,839 ms), *t*_(31)_ = −7.32, *p* < 0.001. However as mentioned above, there is no other factor having a significant effect on participants' reaction time on judging equations. Opposite to our expectations, although statistically non-significant, participants reacted to subtractions following EPUQs (2,844 ms) slower than to subtractions following ENUQs (2,835), *t*_(31)_ = 0.22, *p* = 0.82. On the other hand, participants did react faster to additions following ENUQs (2,528 ms) compared to additions following EPUQs (2,584 ms). However, the results are once again statistically non-significant *t*_(31)_ = 1.44, *p* = 0.16. Figure 2 presents participants' reaction time to the equations in each condition.

Next we analyzed participants' reaction times to sentences as a function of the equation in their preceding trial. The fitted linear model on the data revealed no significant effect of equation type on participants' reaction to the following sentence. In fact the only factor affecting participants' reaction time judging the sentences was the sentence type itself (Table 7).

**Table 7:** Linear model results on arithmetic priming language.

**Effect**	**Estimate**	**SE**	**|*t*|**	***p***
Intercept	2441.47	15.28	159.75	< 0.001^***^
Equation	8.65	15.34	0.56	0.57
Sentence Condition	−34.84	15.34	−2.27	0.02^*^
Equ-Sentence Cond	−16.51	15.34	−1.08	0.28

* *Indicates significance level*.

**Figure 3:**
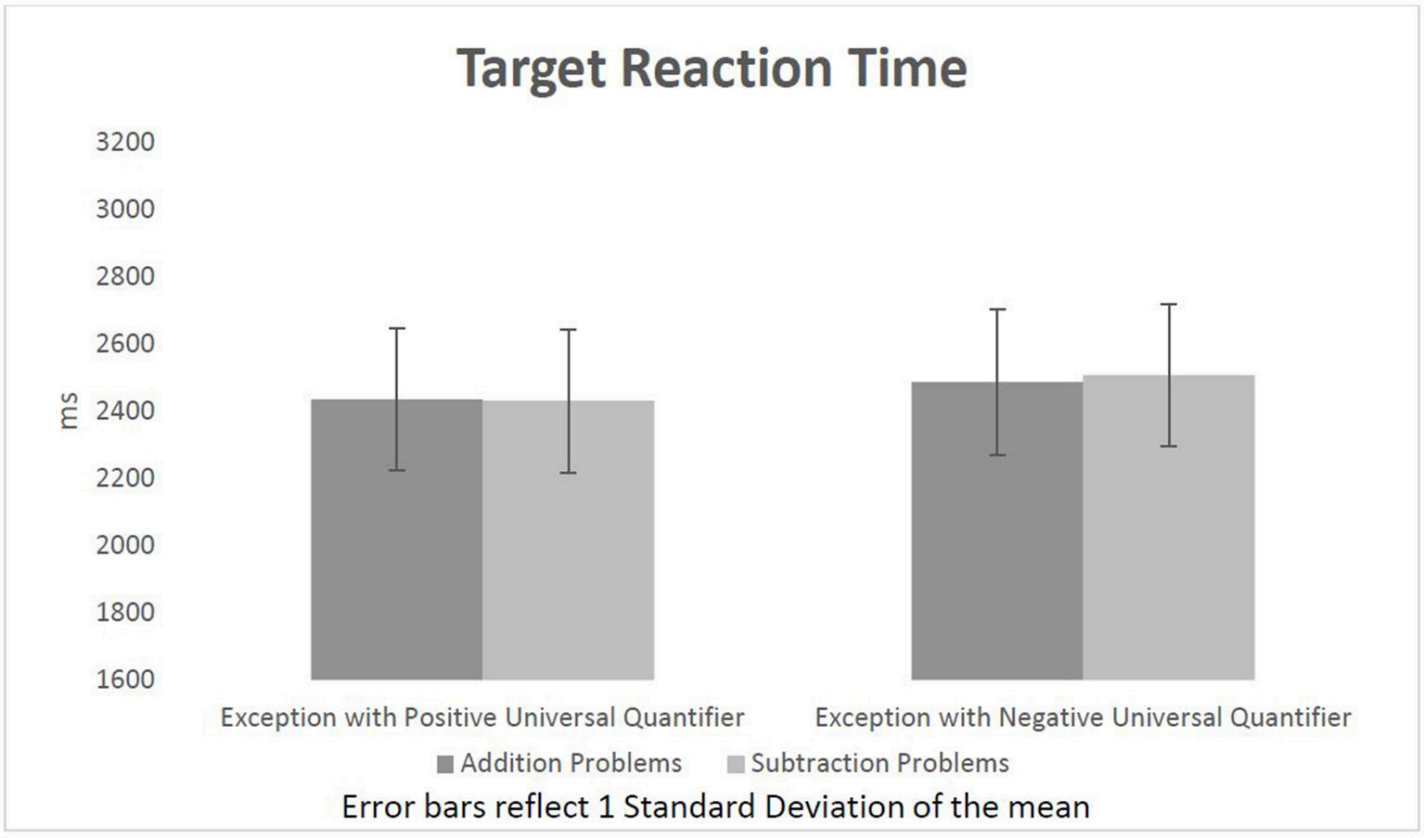
Target reaction time per condition (reverse direction analysis).

According to further *t*-tests, participants reacted significantly faster to sentences with PUQs (2,434 ms) than to the ones with NUQs (2500 ms), *t*_(31)_ = −2.08, *p* = 0.05. The *t*-tests show that EPUQs following subtractions do elicit faster reaction times (2,432 ms) than EPUQs following additions (2,436 ms); however, the effect is not statistically significant *t*_(31)_ = −0.1, *p* = 0.92. Also, ENUQs following additions elicited faster reaction times (2,487 ms) than the ones following subtractions (2,508 ms). This effect is statistically non-significant as well *t*_(31)_ = 0.44, *p* = 0.66. Overall, the data failed to show any significant priming affect in either direction. Figure 3 shows participants' reaction times to sentences in each condition.

Considering the accuracy data in both experiments, we found no reliable differences with the exception of the contrast between EPUQs following subtractions (96%) and EPUQs following additions (98%), *t*_(31)_ = 2.1, *p* < 0.05, supporting the absence of priming benefits.

### Discussion

Improving the stimuli and overcoming the previous limitations caused more accurate reactions by participants judging the sentences. However, the second experiment still shows no significant evidence of cross-domain semantic priming between positive and negative exception sentences, on the one hand, and subtractions or additions, on the other. Given this replication of our null result we are now confident to discuss implications of our findings more generally.

## General discussion

Evidence from structural priming shows that different cognitive modules, such as music, language, and arithmetic share similar networks for integrating and processing syntactic information (Patel, 2003; Scheepers et al., 2011; Scheepers and Sturt, 2014; Van de Cavey and Hartsuiker, 2016). Furthermore, recent semantic priming studies have shown similar evidence for shared semantic processing resources between language and arithmetic, more precisely between semantic categories and addition and division equations (Bassok et al., 2008; Guthormsen et al., 2015). The current experiments were designed to study whether a similar semantic priming effect can be observed between exception sentences and subtraction and addition equations. In order to assess this question, we constructed different priming experiments in which exception sentences acted as primes to addition and subtraction target equations. The design would enable us to study the possibility of a reverse priming effect as well, i.e., whether the equations prime exception sentences.

Two experiments failed to find cross-domain semantic priming between the interpretation of natural language expressions and arithmetic. This could be taken to mean that exception phrases and subtraction/ addition equations are not semantically equivalent after all, even though the semantic literature reviewed in the Introduction defines positive and negative EPs as equivalent to subtractions and additions, respectively. After all, our brain may still process EPs with a different semantic framework, entirely separate from that of mathematical subtractions. In that case EPs would not prime our brain's processing of subtractions or additions.

Another reason for the present lack of cross-domain semantic priming could lie in the individual arithmetic strategies employed by individual participants. It is well-known that some individuals may solve subtraction problems by changing them into their corresponding additions (Campbell, 2008). Therefore, processing EPs and subtraction/ addition equations may rely on shared networks after all, yet this is not revealed by our experiments due to participants using different techniques.

Moreover, admittedly, the specific materials we used in the present study had not been validated for within-domain priming effects. Also, we lost a lot of data through the data trimming phase which could have reduced the power of our statistics. Therefore, it is conceivable that the absence of cross-domain priming effects reflects limitations of these stimuli and number of trials valid for the analysis. Our exclusion criteria were all motivated by rational argument but the relatively large number of excluded trials reduced the statistical power of our approach.

In order to firmly establish the absence of cross-domain semantic priming between exception phrases and either addition or subtraction it will be useful to conduct a follow-up study in which the stimuli is first piloted in a within domain priming experiment. Once each group of stimuli, arithmetic or linguistic, show priming effect in a within domain priming study, we could use them in a cross domain priming experiment to study the relevant processing networks. Furthermore, most of the studies addressed in the literature above use other experimental tasks which engage the participants more actively than a judgment task measuring their accuracy level and reaction time. Therefore, to achieve more accurate results one could conduct an off-line study with a similar design in which participants have to actively calculate and write down the results of the presented addition and subtraction problems. Additionally, participants can be requested to think aloud while solving the arithmetic problems to further group and filter different arithmetic strategies, thus avoiding misguided results due to different arithmetic strategies.

Another factor which may contribute to our results could be the length of our stimuli. While other experiments investigating semantic cross domain priming use short linguistic stimuli (word level), our linguistic stimuli was presented in sentence level. Another improvement for a future study could be limiting the linguistic stimuli to only EPs (e.g., “Jedes Kind außer dem Tisch” instead of “Jedes Kind ist hungrig außer dem Tisch”). As shorter stimuli will also convert to a shorter experiment, we can further increase the number of stimuli to obtain a higher statistic power.

## Ethics statement

This study was carried out in accordance with the recommendations of the British Psychological Society (BPS). All subjects gave their informed consent in accordance with the Declaration of Helsinki. Ethical guidelines for the use of human subjects were strictly followed and the procedure was consistent with the guidelines of the local ethics committee.

## Author contributions

All authors listed have made a substantial, direct and intellectual contribution to the work, and approved it for publication.

### Conflict of interest statement

The authors declare that the research was conducted in the absence of any commercial or financial relationships that could be construed as a potential conflict of interest.
