# Supplementary material for: Language and Arithmetic: A Failure to Find Cross Cognitive Domain Semantic Priming Between Exception Phrases and Subtraction or Addition
Source: Front Psychol. 2018 Aug 23;9:1524. doi: 10.3389/fpsyg.2018.01524 (PMC6116885; doi:10.3389/fpsyg.2018.01524)
Supplement: Supplementary file 1 [file Table_1.pdf]

Appendices for Ronasi et al., Language and Arithmetic

Appendix I: Sentences (Experiment I)

Meaningful Exception Sentences

Alle Frauen außer Katharina kaufen Blumen.
Jedes Kind außer Peter ist hungrig.
Jeder Mann außer Olli isst Burger.
Alle Studenten außer Olli lernen viel
Jede Kuh außer Linda frisst Gras.
Alle Läufer außer Franz haben Wunden.
Alle Angestellten außer Vera bringen Kuchen.
Jede Sängerin außer Jennifer kennt Opern.
Alle Kinder außer Anna haben Bauchschmerzen.
Alle Sportarten außer Ballett sind anstrengend.
Alle Sänger außer Klaus singen gut.
Jede Schülerin außer Mia trägt Hosen.
Jedes Kind außer Lisa backt Kuchen.
Jedes Osterei außer Klaras ist bunt.
Alle Vögel außer Hühner fliegen hoch.
Jeder Student außer Michael fährt Fahrrad.
Jede Farbe außer Schwarz leuchtet schön.
Alle Speisen außer Suppe sind kalt.
Jedes Kind außer Florian spielt draußen.
Alle Mädchen außer Anna machen Sport.
Jede Pianistin außer Carla trinkt Wein.
Jeder Schriftsteller außer Hans ist chaotisch.
Alle Marienkäfer außer Justus sind rot.
Jeder Professor außer Herbert raucht Zigarren.
Alle Freundinnen außer Veronika besitzen Autos.
Jeder Mensch außer Franziska trinkt Milch.
Alle Bauern außer Fritz füttern Schweine.
Jeder Chef außer Papa diktiert Briefe.
Alle Frauen außer Fabienne stricken Strümpfe.
Alle Freunde außer Fiete kommen pünktlich.

Meaningful Non-exception

Alle Frauen kaufen Blumen zum Geburtstag.
Jedes Kind ist hungrig am Abend.
Jeder Mann isst Burger sehr gerne.
Alle Studenten lernen viel für Klausuren.
Jede Kuh frisst Gras auf der Weide.
Alle Läufer haben Wunden am Knie.
Alle Angestellten bringen Kuchen ins Büro.
Jede Sängerin kennt Opern von Wagner.
Alle Kinder haben Bauchschmerzen vom Essen.
Alle Sportarten sind anstrengend in Deutschland.
Alle Sänger singen gut in Konzerten.
Jede Schülerin trägt Hosen mit Knöpfen.
Jedes Kind backt Kuchen mit Schokolade.
Jedes Osterei ist bunt mit Streifen.
Alle Vögel fliegen hoch bei Sonnenschein.
Jeder Student fährt Fahrrad am Wochenende.
Jede Farbe leuchtet schön bei Tageslicht.
Alle Speisen sind kalt am Buffet.
Jedes Kind spielt draußen im Garten.
Alle Mädchen machen Sport im Winter.
Jede Pianistin trinkt Wein zur Pizza.
Jeder Schriftsteller ist chaotisch im Arbeitszimmer.
Alle Marienkäfer sind rot mit Punkten.
Jeder Professor raucht Zigarren im Büro.
Alle Freundinnen besitzen Autos für die Arbeit.
Jeder Mensch trinkt Milch von Kühen.
Alle Bauern füttern Schweine im Stall.
Jeder Chef diktiert Briefe an die Verwaltung.
Alle Frauen stricken Strümpfe am Abend.
Alle Freunde kommen pünktlich zum Treffpunkt.

Meaningless Exception

Alle Frauen außer Katharina kaufen Tod.
Jedes Kind außer Peter ist erwachsen.
Jeder Mann außer Olli isst Bücher.
Alle Studenten außer Olli lernen Flügel.
Jede Kuh außer Linda frisst Koffer.
Alle Läufer außer Franz haben Federn.
Alle Angestellten außer Vera bringen grün.
Jede Sängerin außer Jennifer kennt fliegen.
Alle Kinder außer Anna haben Spitze.
Alle Sportarten außer Ballett sind blau.
Alle Sänger außer Klaus singen Braun.
Jede Schülerin außer Mia trägt Bettdecke.

Meaningless Non-exception

Alle Frauen kaufen Blumen zum Pferd.
Jedes Kind ist hungrig an der Wand.
Jeder Mann isst Burger sehr hell.
Alle Studenten lernen viel für Party.
Jede Kuh frisst Gras auf dem Asphalt.
Alle Läufer haben Wunden am Kühlschrank.
Alle Angestellten bringen Kuchen in Bären.
Jede Sängerin kennt Opern von der Wasserflasche.
Alle Kinder haben Bauchschmerzen vom sehen.
Alle Sportarten sind anstrengend im Schlaf.
Alle Sänger singen gut im Dreieck.
Jede Schülerin trägt Hosen mit Fisch.

Jedes Kind außer Lisa backt Jacke.
Jedes Osterei außer Klaras ist krank.
Alle Vögel außer Hühner fliegen rot.
Jeder Student außer Michael fährt Wasserfall.
Jede Farbe außer Schwarz leuchtet dunkel.
Alle Speisen außer Suppe sind lesbar.
Jedes Kind außer Florian spielt Kreis.
Alle Mädchen außer Anna machen Sonne.
Jede Pianistin außer Carla trinkt Pizza.
Jeder Schriftsteller außer Hans ist quadratisch.
Alle Marienkäfer außer Justus sind Öl.
Jeder Professor außer Herbert raucht Milch.
Alle Freundinnen außer Veronika besitzen Kiemen.
Jeder Mensch außer Franziska trinkt Stein.
Alle Bauern außer Fritz füttern Tische.
Jeder Chef außer Papa diktiert Karton.
Alle Frauen außer Fabienne stricken Nase.
Alle Freunde außer Fiete kommen bewölkt.

Jedes Kind backt Kuchen mit Stift.
Jedes Osterei ist bunt mit Steuer.
Alle Vögel fliegen hoch bei Blumen.
Jeder Student fährt Fahrrad auf dem Wasser.
Jede Farbe leuchtet schön bei Topf.
Alle Speisen sind kalt am Feuer.
Jedes Kind spielt draußen in der Schublade.
Alle Mädchen machen Sport im Schrank.
Jede Pianistin trinkt Wein zum Stern.
Jeder Schriftsteller ist chaotisch im Himmel.
Alle Marienkäfer sind rot mit Wolken.
Jeder Professor raucht Zigarren im Steak.
Alle Freundinnen besitzen Autos für den Schlaf.
Jeder Mensch trinkt Milch von Fotos.
Alle Bauern füttern Schweine im Badezimmer.
Jeder Chef diktiert Briefe an die Biene.
Alle Frauen stricken Strümpfe unter Autos.
Alle Freunde kommen pünktlich zur Sahne.

Appendix II: Equations (Experiments I & II)

Correct Additions	Incorrect Additions	Correct Subtractions	Incorrect Subtractions
33+8=41	33+9=44	11-5=6	28-9=17
22+9=31	19+8=25	11-7=4	31-5=28
35+8=43	34+9=45	31-7=24	11-9=4
23+9=32	23+8=33	31-9=22	12-4=6
35+9=44	24+9=35	12-6=6	32-4=26
25+8=33	36+5=43	12-7=5	32-6=28
25+9=34	36+6=44	33-5=28	32-7=26
36+9=45	26+5=33	14-5=9	13-5=6
26+9=35	26+6=34	14-8=6	13-6=5
37+4=41	37+5=44	34-5=29	33-6=29
27+5=32	27+4=33	35-6=29	15-6=7
37+7=44	37+6=41	17-9=8	34-8=28
37+8=45	27+6=31	35-7=28	15-7=6
27+7=34	38+3=43	21-5=16	37-9=26
27+8=35	38+4=44	33-4=29	21-7=16
38+5=43	28+3=33	22-4=18	34-7=25
38+6=44	38+7=47	22-6=16	21-9=14
28+4=32	28+5=31	36-8=28	36-7=27
28+7=35	38+8=48	41-5=36	38-9=27
39+2=41	28+6=33	23-6=17	22-7=17
28+8=36	39+4=41	41-9=32	23-5=16
39+3=42	29+2=33	24-5=19	41-7=36
39+5=44	29+3=34	42-4=38	24-8=14
29+4=33	39+6=43	13-4=9	25-6=17
29+5=34	29+6=37	25-7=18	42-6=38
39+7=46	39+8=49	27-9=18	42-7=36
29+7=36	42+9=53	14-7=7	23-4=17
39+9=48	29+8=35	26-7=19	24-7=15
43+8=51	43+9=54	18-9=9	16-7=7
29+9=38	32+9=43	26-8=18	16-8=6

Appendix III: Sentences (Experiment II)

Meaningful Positive Universal
Alle Frauen kaufen Blumen, außer Katharina.
Jedes Kind ist hungrig, außer Peter.
Jeder Mann isst Burger, außer Olli.
Alle Studenten lernen viel, außer Olli.
Jede Kuh frisst Gras, außer Linda.
Alle Läufer haben Wunden, außer Franz.
Alle Angestellten bringen Kuchen, außer Vera.
Jede Sängerin kennt Opern, außer Jennifer.
Alle Kinder haben Bauchschmerzen, außer Anna.
Alle Sportarten sind anstrengend, außer Ballett.
Alle Sänger singen gut, außer Klaus.
Jede Schülerin trägt Hosen, außer Mia.
Jedes Kind backt Kuchen, außer Lisa.
Jedes Osterei ist bunt, außer Klaras.
Alle Vögel fliegen hoch, außer Hühner.
Jeder Student fährt Fahrrad, außer Michael.
Jede Farbe leuchtet schön, außer Schwarz.
Alle Speisen sind kalt, außer der Suppe.
Jedes Kind spielt draußen, außer Florian.
Alle Mädchen machen Sport, außer Anna.
Jede Pianistin trinkt Wein, außer Carla.
Jeder Schriftsteller ist chaotisch, außer Hans.
Alle Marienkäfer sind rot, außer Justus.
Jeder Professor raucht Zigarren, außer Herbert.
Alle Freundinnen besitzen Autos, außer Veronika.
Jeder Mensch trinkt Milch, außer Franziska.
Alle Bauern füttern Schweine, außer Fritz.
Jeder Chef diktiert Briefe, außer Papa.
Alle Frauen stricken Strümpfe, außer Fabienne.
Alle Freunde kommen pünktlich, außer Fiete.

Meaningful Negative Universal
Keine Frauen kaufen Blumen, außer Katharina.
Kein Kind ist hungrig, außer Peter.
Kein Mann isst Burger, außer Olli.
Keine Studenten lernen viel, außer Olli.
Keine Kuh frisst Gras, außer Linda.
Keine Läufer haben Wunden, außer Franz.
Keine Angestellten bringen Kuchen, außer Vera.
Keine Sängerin kennt Opern, außer Jennifer.
Keine Kinder haben Bauchschmerzen, außer Anna.
Keine Sportarten sind anstrengend, außer Ballett.
Keine Sänger singen gut, außer Klaus.
Keine Schülerin trägt Hosen, außer Mia.
Kein Kind backt Kuchen, außer Lisa.
Kein Osterei ist bunt, außer Klaras.
Keine Vögel fliegen hoch, außer Hühner.
Kein Student fährt Fahrrad, außer Michael.
Keine Farbe leuchtet schön, außer Schwarz.
Keine Speisen sind kalt, außer der Suppe.
Kein Kind spielt draußen, außer Florian.
Keine Mädchen machen Sport, außer Anna.
Keine Pianistin trinkt Wein, außer Carla.
Kein Schriftsteller ist chaotisch, außer Hans.
Keine Marienkäfer sind rot, außer Justus.
Kein Professor raucht Zigarren, außer Herbert.
Keine Freundinnen besitzen Autos, außer Veronika.
Kein Mensch trinkt Milch, außer Franziska.
Keine Bauern füttern Schweine, außer Fritz.
Kein Chef diktiert Briefe, außer Papa.
Keine Frauen stricken Strümpfe, außer Fabienne.
Keine Freunde kommen pünktlich, außer Fiete.

Meaningless Positive Universal
Alle Frauen kaufen Blumen, außer Jens.
Jedes Kind ist hungrig, außer dem Tisch.
Jeder Mann isst Burger, außer der Lampe.
Alle Studenten lernen viel, außer der Tasse.
Jede Kuh frisst Gras, außer der Katze.
Alle Läufer haben Wunden, außer der Tür.
Alle Angestellten bringen Kuchen, außer dem Koffer.
Jede Sängerin kennt Opern, außer dem Kasten.
Alle Kinder haben Bauchschmerzen, außer dem Sessel.
Alle Sportarten sind anstrengend, außer dem Teppich.
Alle Sänger singen gut, außer dem Glas.
Jede Schülerin trägt Hosen, außer dem Himmel.
Jedes Kind backt Kuchen, außer dem Fenster.

Meaningless Negative Universal
Keine Frauen kaufen Blumen, außer Jens.
Kein Kind ist hungrig, außer dem Tisch.
Kein Mann isst Burger, außer der Lampe.
Keine Studenten lernen viel, außer der Tasse.
Keine Kuh frisst Gras, außer der Katze.
Keine Läufer haben Wunden, außer der Tür.
Keine Angestellten bringen Kuchen, außer dem Koffer.
Keine Sängerin kennt Opern, außer dem Kasten.
Keine Kinder haben Bauchschmerzen, außer dem Sessel.
Keine Sportarten sind anstrengend, außer dem Teppich.
Keine Sänger singen gut, außer dem Glas.
Keine Schülerin trägt Hosen, außer dem Himmel.
Kein Kind backt Kuchen, außer dem Fenster.

Jedes Osterei ist bunt, außer dem Hähnchen.
Alle Vögel fliegen hoch, außer der Giraffe.
Jeder Student fährt Fahrrad, außer dem Vorhang.
Jede Farbe leuchtet schön, außer dem Wald.
Alle Speisen sind kalt, außer der Decke.
Jedes Kind spielt draußen, außer dem Nachtisch.
Alle Mädchen machen Sport, außer der Flasche.
Jede Pianistin trinkt Wein, außer dem Buch.
Jeder Schriftsteller ist chaotisch, außer dem Baum.
Alle Marienkäfer sind rot, außer der Fabrik.
Jeder Professor raucht Zigarren, außer der Maus.
Alle Freundinnen besitzen Autos, außer der Wolke.
Jeder Mensch trinkt Milch, außer dem Dampf.
Alle Bauern füttern Schweine, außer der Brücke.
Jeder Chef diktiert Briefe, außer dem Eis.
Alle Frauen stricken Strümpfe, außer dem Strand.
Alle Freunde kommen pünktlich, außer dem Bleistift.

Kein Osterei ist bunt, außer dem Hähnchen.
Keine Vögel fliegen hoch, außer der Giraffe.
Kein Student fährt Fahrrad, außer dem Vorhang.
Keine Farbe leuchtet schön, außer dem Wald.
Keine Speisen sind kalt, außer der Decke.
Kein Kind spielt draußen, außer dem Nachtisch.
Keine Mädchen machen Sport, außer der Flasche.
Keine Pianistin trinkt Wein, außer dem Buch.
Kein Schriftsteller ist chaotisch, außer dem Baum.
Keine Marienkäfer sind rot, außer der Fabrik.
Kein Professor raucht Zigarren, außer der Maus.
Keine Freundinnen besitzen Autos, außer der Wolke.
Kein Mensch trinkt Milch, außer dem Dampf.
Keine Bauern füttern Schweine, außer der Brücke.
Kein Chef diktiert Briefe, außer dem Eis.
Keine Frauen stricken Strümpfe, außer dem Strand.
Keine Freunde kommen pünktlich, außer dem Bleistift.
